# Isolation, Characterization and Biological Evaluation of Jellyfish Collagen for Use in Biomedical Applications

**DOI:** 10.3390/md9060967

**Published:** 2011-06-07

**Authors:** Sourour Addad, Jean-Yves Exposito, Clément Faye, Sylvie Ricard-Blum, Claire Lethias

**Affiliations:** 1 Université Lyon 1, Univ Lyon, CNRS, FRE 3310, Dysfonctionnement de l’Homéostasie Tissulaire et Ingénierie Thérapeutique, IBCP, 7 passage du Vercors, F-69367, France; E-Mails: sourouraddad@yahoo.fr (S.A.); jy.exposito@ibcp.fr (J.-Y.E.); 2 Université Lyon 1, Univ Lyon, CNRS, UMR 5086, Bases Moléculaires et Structurales des Systèmes Infectieux, IBCP 7 passage du Vercors, F-69367, France; E-Mails: clementfaye1@yahoo.fr (C.F.); s.ricard-blum@ibcp.fr (S.R.-B.)

**Keywords:** collagen, jellyfish, biocompatibility, cell adhesion, cross-linking

## Abstract

Fibrillar collagens are the more abundant extracellular proteins. They form a metazoan-specific family, and are highly conserved from sponge to human. Their structural and physiological properties have been successfully used in the food, cosmetic, and pharmaceutical industries. On the other hand, the increase of jellyfish has led us to consider this marine animal as a natural product for food and medicine. Here, we have tested different Mediterranean jellyfish species in order to investigate the economic potential of their collagens. We have studied different methods of collagen purification (tissues and experimental procedures). The best collagen yield was obtained using *Rhizostoma pulmo* oral arms and the pepsin extraction method (2–10 mg collagen/g of wet tissue). Although a significant yield was obtained with *Cotylorhiza tuberculata* (0.45 mg/g), *R. pulmo* was used for further experiments, this jellyfish being considered as harmless to humans and being an abundant source of material. Then, we compared the biological properties of *R. pulmo* collagen with mammalian fibrillar collagens in cell cytotoxicity assays and cell adhesion. There was no statistical difference in cytotoxicity (*p* > 0.05) between *R. pulmo* collagen and rat type I collagen. However, since heparin inhibits cell adhesion to jellyfish-native collagen by 55%, the main difference is that heparan sulfate proteoglycans could be preferentially involved in fibroblast and osteoblast adhesion to jellyfish collagens. Our data confirm the broad harmlessness of jellyfish collagens, and their biological effect on human cells that are similar to that of mammalian type I collagen. Given the bioavailability of jellyfish collagen and its biological properties, this marine material is thus a good candidate for replacing bovine or human collagens in selected biomedical applications.

## 1. Introduction

Collagens are often considered as an animal hallmark [1]. They form an abundant structural protein family that is widely represented throughout the tissues of the body. All collagen molecules contain a triple-helical domain, are generally involved in the formation of supramolecular networks, and are made of three α chains which may or may not be identical [2]. These α chains contain at least one collagenous domain or triple helix motif characterized by the succession of Gly-Xaa-Yaa triplets where Xaa and Yaa are often Pro and Hyp residues, respectively. Association of the collagenous region of the three α chains allows the formation of the triple-helical domain. Although numerous collagen families have been characterized in all Metazoa, only two of them are present from sponges to human, *i.e.*, the fibrillar and the basement membrane type IV collagens [3,4]. The fibrillar collagens form the well-known striated-fibrils. Their α chain precursors contain a large uninterrupted collagenous domain made of approximately 338 Gly-Xaa-Yaa triplets, this region being flanked by two non-collagenous sequences, the *N*- and the *C*-propeptides. These propeptides are generally removed by specific proteinases during the maturation of procollagens into collagen molecules, before the formation of the D-staggered collagen fibrils.

Collagen fibrils have important structural functions in the mechanical properties of tissues like tendons, skin and bone. They also are involved in numerous biological functions, from the early stages of development to tissue repair. In addition to these characteristics, their biodegradability and their poor immunogenicity explain that these proteins have an important economic impact. They have been used as gelatin (boiled collagens representing denatured molecules) in the food industry, and as leather. Collagens also are widely used in the cosmetic and pharmaceutical industries [5]. During the last decades, safety problems like the mad cow disease incident (bovine spongiform encephalopathy) induced the development of alternatives to bovine collagen. One area of research has been the production of recombinant collagens in different host models like bacteria, mouse milk, plants or yeast [6–9]. An alternative approach has been to look at marine resources, and for example to extract collagen from waste (bone, scale and skin) products from fishing companies [10,11]. Most marine animals are invertebrates. From genomic programs, molecular cloning, biochemical and/or ultrastructural studies, it has been demonstrated that invertebrate fibrillar collagens share the same characteristics than their human counterparts [12]. In agreement with this, new industrial prospects might be to choose a system that: (1) avoids mammalian tissues, (2) is useful in terms of CO_2_ emission and (3) takes into account the management of natural wastes and/or ecological problems.

Under these conditions, jellyfish might be a model of interest. They are often considered as gelatinous animals (mostly water and a developed collagen-rich mesogloea), and their increasingly frequent outbreaks generate ecological and economic consequences from the formation of ocean jellyfish to beach closures [13]. The goal of this study is to characterize jellyfish collagen to evaluate its use as a source of marine collagen to prepare implantable biomaterials for humans. Here, we have compared the potential use of different jellyfish species collected near the Tunisian coasts (Mediterranean Sea), and frozen before the extraction of fibrillar collagens. We analyzed their biochemical and their biological characteristics, and compared these data to previous data concerning jellyfish collagens.

## 2. Results and Discussion

### 2.1. Jellyfish Collagen Purification

Specimens from four jellyfish species (*Aurelia aurita*, *Cotylorhiza tuberculata*, *Pelagia noctiluca*, and *Rhizostoma pulmo*) were collected from Tunisian Mediterranean coast, and frozen after being caught. In order to estimate the potential of these jellyfish species as a source of collagen, we have tested different collagen extraction procedures from several tissues. The extraction yields of acid-soluble and pepsinized collagens from umbrella (exumbrella plus subumbrella), oral arms, or whole animal for *P. noctiluca* and *A. aurita* (the anatomy of these two species was not preserved during the freeze-thaw procedure), are presented in Table 1. The lowest yields were obtained with the acid-soluble extraction method, and when the extraction was carried out on whole tissues. The best yield was obtained from *R. pulmo* oral-arms (Table 1, 2.61 to 10.3 mg/g). A good extraction yield was also obtained for *C. tuberculata*, but its limited bioavailability in the Tunis Bay [14] in comparison to *R. pulmo* led us to select pepsin-soluble collagen extracted from *R. pulmo* oral arms for further studies.

**Figure 1:**
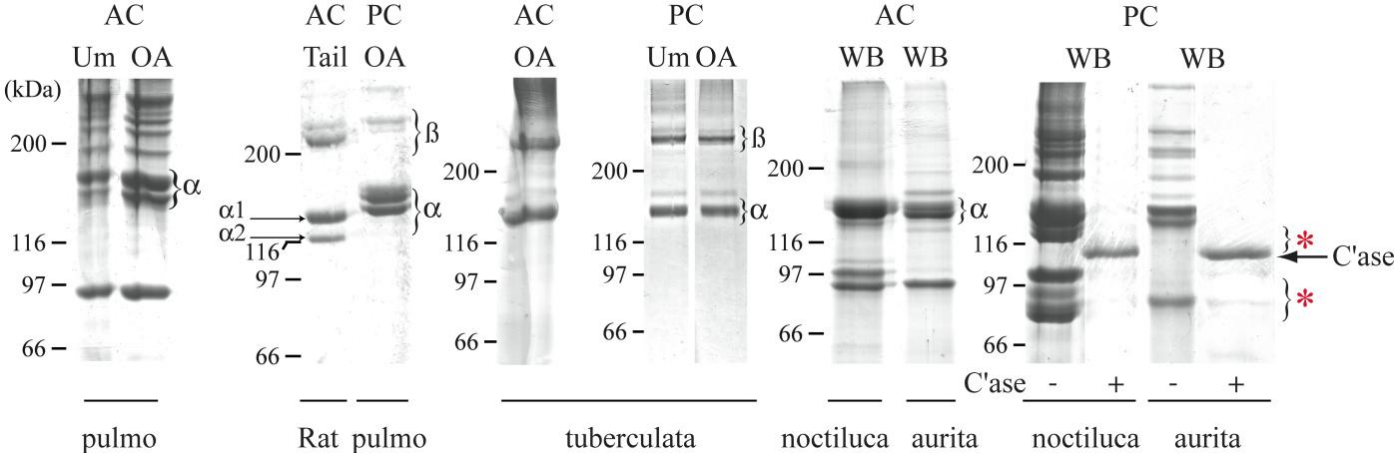
SDS-PAGE analysis of jellyfish collagens. Acid-soluble (AC) and pepsinized (PC) collagens of *R. pulmo* (pulmo), *C. tuberculata* (tuberculata), *P. noctiluca* (noctiluca) and *A. aurita* (aurita) were loaded on 6% polyacrylamide gels. AC tail rat type I collagen was used as fibrillar collagen control. The jellyfish fibrillar α chains (α) and dimers of cross-linked α chains (β) were indicated. Jellyfish collagens have been extracted from umbrella (Um), oral arms (OA) or whole body (WB). The red asterisks denote putative degraded products. C’ase: collagenase. The positions of molecular mass markers (kDa) are indicated on the left of the gels.

SDS-PAGE analysis of collagenous extracts was presented in Figure 1. It should be noted that triple-helical proteins have an apparent electrophoretic mobility in SDS-PAGE that is not correlated to their molecular masses due to their low content of hydrophobic amino acid residues [15,16]. For all the jellyfish collagen extracts, the bands corresponding to the collagen α chains have an apparent molecular mass similar or slightly higher than the rat α1(I) chain (Figure 1). With the exception of *C. tuberculata* samples, the patterns of α chains in the pepsinized extracts are more complex than in acid-soluble samples. These differences are evident in the presence of additional and faster migrating chains, highlighted by asterisks in Figure 1. These bands being collagenase-sensitive (Figure 1), they could represent an over-pepsinization of the collagen chains. Similar results have been reported for another jellyfish species [17]. These degradation products might correspond to the presence of less folded and thermally unstable regions in these collagen molecules, which are more sensitive to protease digestion. Indeed, it has been shown that proline residues, and more precisely hydroxyproline residues, play a crucial role in the stability of the triple helical structure [18], and that jellyfish collagens [17,19,20] contain less imino acid residues and a lower melting temperature (122 to 142‰ and 29 °C) than mammalian type I fibrillar collagen (approximately 220‰ and 37–41 °C) [21]. This is in agreement with the fact that invertebrate fibrillar α chains are usually poorer in proline residues than mammalian collagens [1]. Hence, in the sea anemone *Nematostella vectensis*, a representative of the anthozoan class of Cnidaria, we have previously identified the primary structure of eight fibrillar α chains containing between 120 and 180‰ of proline in the collagenous sequence [22].

The best collagen yields have been obtained from *R. pulmo* oral arms by the pepsin-extraction method. *R. pulmo*, the largest jellyfish species tested in our study, is moderately venomous and is generally considered harmless to humans [14]. For these reasons, pepsin-soluble collagen extracted from *R. pulmo* oral arms was selected for further studies.

### 2.2. *R. pulmo* Collagen Stability

**Figure 2:**
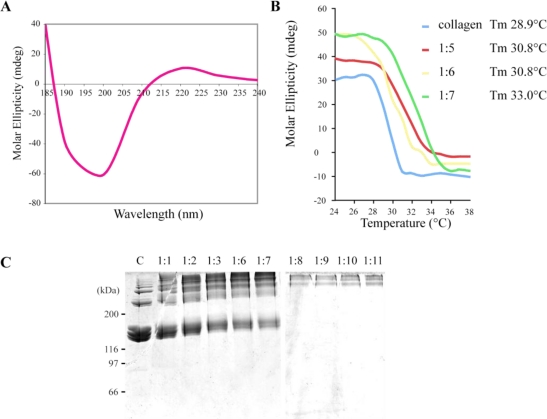
Melting temperature of pepsinized and EDC cross-linked *R. pulmo* collagens. (**A**) Circular dichroism spectra recorded at 25 °C for pepsinized collagen; (**B**) Thermal transition curves of pepsinized or EDC cross-linked (1:5 to 1:7 collagen/EDC ratios, w/w) collagens (300 μg/mL) were monitored by circular dichroism at 220 nm through 0.5 °C/min ramps (from 22 to 40 °C). Melting temperature (Tm) for each condition is indicated in the legend; (**C**) SDS/PAGE analysis of EDC cross-linked jellyfish collagens. Different collagen/EDC ratios (w/w) were loaded and compared to untreated collagen (C, control).

Heat stability and cross-linking of collagen molecules are important features for their use as biomaterials [23]. The melting temperature of *R. pulmo* collagen calculated from circular dichroism data was 28.9 °C (Figure 2A,B). In order to stabilize collagen and to obtain a jellyfish collagen with a melting temperature closest to that of mammalian type I collagens, by cross-linking, we have used the non-hazardous, water-soluble chemical cross-linker 1-ethyl-3-(3-dimethylaminopropyl)carbodiimide hydrochloride (EDC). As shown in Figure 2C, increase in the EDC/collagen ratio against collagen increased the formation of high molecular mass products (>200 kDa) indicating that cross-linking has occurred. EDC treatment increased the melting temperature of *R. pulmo* collagen by several degrees as shown by circular dichroism. For a collagen/EDC ratio of 1:7, the melting temperature was 33 °C instead of 28.9 °C for the non-cross-linked collagen (Figure 2B). The formation of aggregates at higher collagen/EDC ratios prevented us to record circular dichroism spectra but melting temperature should further increase in these conditions. Using the same cross-linking agent, Song *et al.* [24] have been able to decrease the enzymatic degradation of jellyfish collagen scaffolds *in vitro*. Thus, this cross-linked method seems to be suitable to modulate the bio-degradability of jellyfish collagen. Although the melting temperature of jellyfish collagen was below human body temperature, it could be used in combination with other polymers such as chitosan to make resorbable biomaterials as reported by Wang *et al.* who developed an injectable chitosan/marine collagen composite gel [25].

### 2.3. Biochemical Characterization of *R. pulmo* Fibrillar Collagen

**Figure 3:**
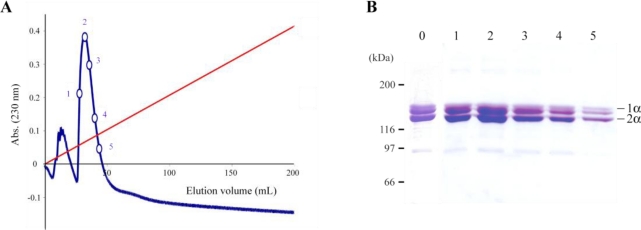
Analysis of native (non-denatured) *R. pulmo* fibrillar collagen. (**A**) CM-cellulose ion chromatography of native collagen. Collagen sample in 0.06 M sodium acetate, pH 4.8, was applied onto the CM-cellulose column. After washing with the same buffer, the elution was performed with a 0–0.5 M linear gradient of NaCl (red line). A flow rate of 40 mL/h was used in this experiment. Numbers indicate the elution fractions analyzed by SDS-PAGE (**B**). Fractions were loaded on 6% acrylamide gel and stained with Coomassie Brilliant Blue R-250. Lane 0: sample before application to the column, lanes 1–5: eluted fractions.

**Figure 4:**
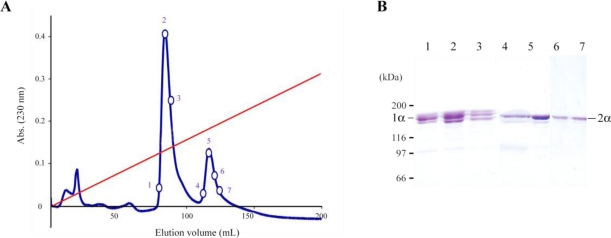
Analysis of denatured *R. pulmo* collagen. (**A**) CM-cellulose chromatography of denatured collagen. Denatured collagen in 0.02 M sodium acetate was subjected to a CM-cellulose chromatography. Elution was realized with a 0–0.15 M linear gradient of NaCl (red line); (**B**) SDS-PAGE of fractions numbered in (A). The migration of *R. pulmo* collagen chains is indicated by 1α and 2α.

Type I collagen/cell interactions are mediated by different types of receptors, like integrins, cell surface heparan sulfate proteoglycans and discoidin domain receptors [26,27]. Before investigating the biological effect of *R. pulmo* collagen on mammalian cells, we have characterized the molecular composition of *R. pulmo* collagen and its heparin-binding site. Native or heat denatured *R. pulmo* collagens were subjected to ion-exchange CM-cellulose chromatography (Figures 3A and 4A, respectively), and each fraction was analyzed by SDS-PAGE (Figures 3B and 4B). For the native collagen sample, one major peak was eluted (Figure 3A), suggesting the presence of a single molecular collagen isoform. SDS-PAGE analysis confirmed that the different fractions had the same pattern that the unfractionated sample (Figure 3B). The presence of a minor peak has been reported by another group [28]. According to these authors the small peak might correspond to small amounts of collagen breakdown products and/or small non-collagenous proteins. However, we failed to detect any protein by Coomassie staining upon analysis of this minor peak by SDS-PAGE (data not shown).

In order to determine its molecular composition jellyfish collagen was chromatographied after heat denaturation that dissociated the triple helix into individual α chains. Two major peaks were observed, suggesting the existence of two different α chains (Figure 4A). According to calculations based on the peak areas (Figure 4A) and to SDS-PAGE analysis (Figure 4B), the molecular composition of *R. pulmo* collagen could be [(1α)_2_2α] (*i.e*., two 1α chains and one 2α chain).

**Figure 5:**
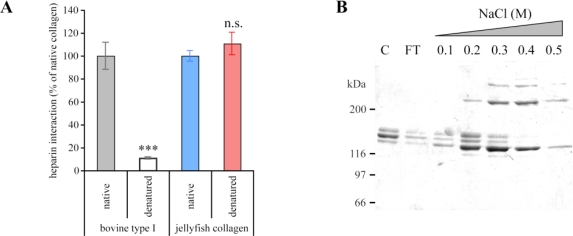
Native and denatured *R. pulmo* collagens interact with heparin. (**A**) Solid phase assays were used to analyze heparin/collagen interactions. Bovine pepsinized collagen was used as a positive control. Native or denatured bovine or jellyfish pepsinized collagens were coated at 5 μg/mL. (***) *p* < 0.001; (n.s.) non significant; Student’s *t*-test; (**B**) Eluted fractions from heparin-affinity chromatography were analyzed by SDS-PAGE. C, Jellyfish collagen before chromatography; FT, flow-through fraction.

As previously shown [29], type I collagen possesses a conformational binding site for heparin. By solid phase assays, we have demonstrated that *R. pulmo* collagen contains at least one heparin-binding site (Figure 5A). Moreover, denatured *R. pulmo* collagen retains its ability to bind to heparin suggesting that the triple helix is not required for the binding and that at least one heparin-binding site is linear. Denatured jellyfish collagen was subjected to heparin affinity chromatography and specific eluted fractions were analyzed by SDS-PAGE (Figure 5B). As shown in Figure 5B, the linear heparin binding site is located within the 2α chain, this result being in agreement to the molecular composition deduced from the CM-cellulose chromatography (Figure 4). However, from the presence of proteolytic fragments (see Figure 1), and from the CM-cellulose results obtained in other studies [19,20], we cannot rule out the hypothesis that the peak containing the 1α chain (Figure 4A) actually contains two different α chains with similar chemical properties.

### 2.4. Biological Properties of *R. pulmo* Fibrillar Collagen

**Figure 6:**
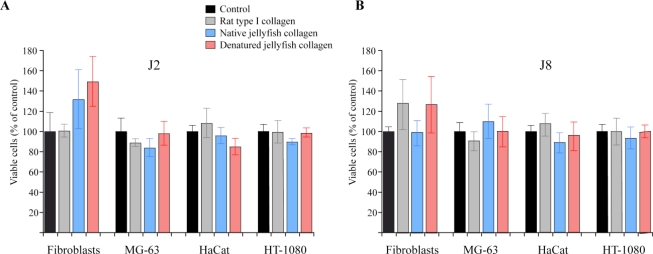
*R. pulmo* collagen did not exhibit cytotoxic activity against different cell lines. Control experiments consist of cells cultured in uncoated wells. Coating was made using native or denatured acid-soluble rat type I and jellyfish collagens at 50 μg/mL. Primary fibroblasts, osteoblastic (MG-63), epithelial (HaCat) and fibrosarcoma (HT-1080) cell lines were cultured for 2 days (**A**) or 8 days (**B**). Student’s *t*-test was performed to compare each coating condition to the uncoated control. The results were not significantly different.

In the perspective to use *R. pulmo* as a natural marine biomaterial, its cytotoxicity and its effect on cell adhesion were investigated. In order to detect a possible toxicity of jellyfish collagen, human cells originating from different tissues were cultured for two or eight days on *R. pulmo* native or denaturated collagen. For all the cell lines tested (fibroblastic, epithelial, osteoblastic and fibrosarcoma), the amount of viable cells on jellyfish collagen-coated wells was not significantly different from rat type I collagen or from uncoated wells (Figure 6). These results are in agreement with the study of Song *et al.* [24], performed with fibroblasts, chondrocytes, endothelial and smooth muscle cells. Taken together, these data confirm the harmlessness of jellyfish collagens in the experimental conditions used.

**Figure 7:**
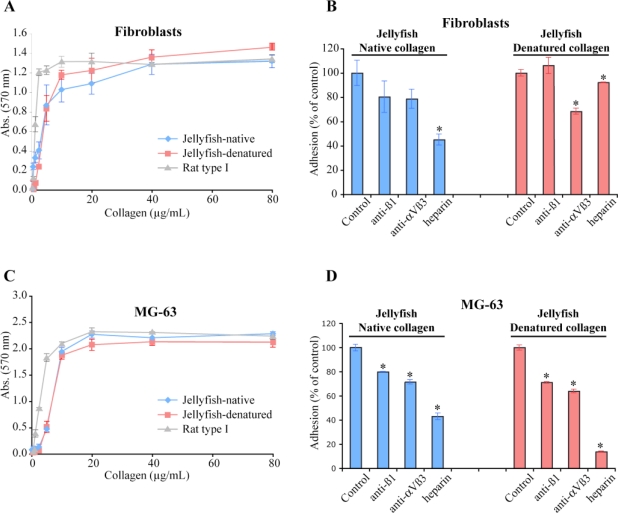
Cellular receptors of *R. pulmo* collagens. Dose-response profiles of human fibroblasts (**A**) and MG-63 (**C**) to native rat type I, and native and denatured jellyfish collagens from *R. pulmo*. Fibroblast (**B**) or MG-63 cell (**D**) adhesion to native or denatured jellyfish collagen in the presence of anti-integrin antibody, heparin, or without inhibitor (control). Cell adhesion was quantified by a colorimetric assay using crystal violet. Values shown are the mean of triplicates minus non-specific binding on BSA, and statistical analyses were carried out from three independent experiments. (*) *p* < 0.05, Student’s *t*-test.

The interaction of fibroblastic and osteoblastic cells with *R. pulmo* collagen was investigated in more details. We quantified cell adhesion and showed that both cell lines efficiently adhere to jellyfish collagen (Figure 7). In these quantitative cell adhesion assays, similar curves were obtained with native or denatured collagen molecules (Figure 7A,C). Cells adhere in a dose dependent-manner, the sub-optimal coating concentration being approximately 10 to 20 μg/mL. In order to identify the cellular receptors involved in the interaction with jellyfish collagen, inhibition studies were performed with antibodies against integrins or with heparin. On native collagen, similar inhibition profiles were observed for osteoblastic cells (MG-63) or fibroblasts. Indeed, cell adhesion was slightly inhibited (20–30%) by function-blocking anti-β1 or anti-αVβ3 integrin antibodies (Figure 7B,D). Using the same experimental approach, more than 90% of adhesion was inhibited when fibroblasts or MG-63 cells were deposited onto rat native type I collagen in the presence of anti-β1 antibody (data not shown). This result is in agreement with data indicating that α1β1, α2β1, α10β1 and α11β1 act as receptors for native mammalian fibrillar collagens [30–32]. Moreover, denaturation of collagens unmasks cryptic RGD sites interacting with αVβ3 integrin [33]. From our experiments on jellyfish collagen, we are able to deduce that αVβ3 and β1-containing integrins are not the major cellular receptors for jellyfish collagen. The slight cell adhesion inhibition observed with anti-αVβ3 in native assays (Figure 7B,D) might be due to the presence of partially folded regions in the jellyfish collagen molecule. However, even on denatured jellyfish collagens, the anti-αVβ3 antibody did not fully block cell adhesion (35–38% of inhibition), suggesting that other receptors participate in the adhesion process.

**Figure 8:**
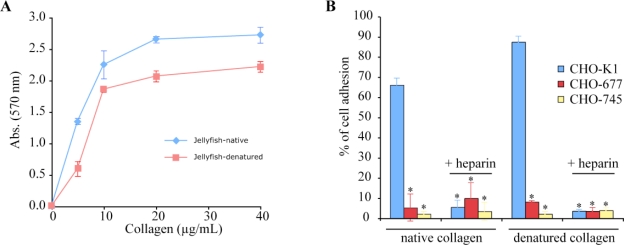
Adhesion of CHO cells to *R. pulmo* collagens: (**A**) Dose-response profiles of CHO-K1 cells to native and denatured jellyfish collagens; (**B**) Adhesion of CHO-K1 or of the mutants (CHO-677 and CHO-745) to native or denatured jellyfish collagens in the presence or absence of heparin. Statistical analyses were performed from three independent experiments. (*) *p* < 0.005, Student’s *t*-test.

In order to test the involvement of cell surface heparan-sulfate chains, we performed cell adhesion inhibition studies with heparin. Indeed, a significant inhibition was obtained on native collagen for both cell lines (55–60%), and for MG-63 cells plated on denatured jellyfish collagen (Figure 7B,D). To confirm these data, we used wild-type and mutant CHO cell lines expressing various levels of glycosaminoglycans at their surface, namely CHO-K1 expressing both heparan and chondroitin sulfates, and the mutant derivatives CHO-677, deficient in heparan sulfate, and CHO-745, that did not synthesize glycosaminoglycans [34,35]. As shown in Figure 8A, CHO-K1 cells exhibited a firm adhesion to both native and denatured jellyfish collagen. This interaction to CHO-K1 cells was almost completely inhibited by heparin, while the mutant cell lines (CHO-677 and CHO-745) did not interact with these collagens (Figure 8B). These results suggest that, in contrast to heparan sulfate proteoglycans, integrins do not contribute significantly to fibroblast adhesion to jellyfish collagen. The adhesion of MG-63 cells to jellyfish collagen is mediated by αVβ3 and β1 integrins and by heparan sulfate proteoglycans.

**Figure 9:**
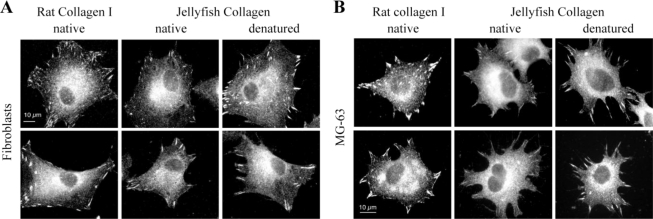
Focal adhesion structures developed in cells adhering to *R. pulmo* collagens. Vinculin immuno-localization in human fibroblasts (**A**) and MG-63 cells (**B**) plated onto native rat type I collagen, and onto native and denatured jellyfish collagens.

Cell adhesion to the extracellular matrix is mediated by focal adhesions, which are specialized structures involved in the coupling of cytoskeletal elements to membrane receptors, and in the recruitment of signaling complexes [36]. The assembly of focal adhesions is considered a relevant test to analyze *in vitro* biocompatibility [37]. We attempted to identify such structures in cells interacting with jellyfish collagen, by immunofluorescent labeling of vinculin. Fibroblasts develop focal adhesion structures when plated on native or denatured *R. pulmo* collagen (Figure 9A). A similar morphology of contacts and cell spreading was observed with fibroblasts adhering to rat type I collagen (Figure 9A). MG-63 cells assemble focal adhesions on jellyfish collagens, but cell spreading seems to be slightly diminished by comparison with their morphology on rat type I collagen (Figure 9B). From these results, we can hypothesize that molecular determinants of collagens involved in cell adhesion are at least partly conserved throughout evolution since human cells adhere to their substrates using integrin and/or heparan sulfate receptors [38,39], and develop cell matrix contacts when plated on jellyfish collagens.

## 3. Experimental Section

### 3.1. Jellyfish Collagen Purification

All collagen purification steps were carried out at 4 °C. Frozen jellyfish tissues were powdered in liquid nitrogen. A minimum of 10 g of tissue was used for extraction in each experiment with 10 mL extraction solution/g tissue. Tissue powders were mixed with 0.5 M acetic acid, and acid-soluble collagens were extracted overnight under continuous stirring. This mixture was then centrifuged (15,000 *g*, 1 h). The pellet was used for further extraction with pepsin (see below), and acid-soluble collagen was precipitated from the supernatant by adjusting the final NaCl concentration to 0.9 M. The resultant precipitate was recovered by centrifugation (15,000 *g*, 1 h). The pellet (acid-soluble collagen) was then resuspended into 0.5 M acetic acid and dialyzed against 0.1 M acetic acid.

The pellet obtained after acid extraction was dissolved in 0.1 M acetic acid, and pepsin was added (2–15 mg pepsin/mg of wet tissue). The digestion mixture was incubated overnight, and pepsin activity was then inhibited by increasing the pH to 6.0–6.5 with NaOH and by adding pepstatin A to a final concentration of 1 μM. After centrifugation (15,000 *g*, 1 h), the supernatant was dialyzed against 20 mM Na_2_HPO_4_. The resulting precipitate (pepsinized-collagen) was collected by centrifugation and then dissolved in 0.5 M acetic acid. The collagen was precipitated by addition of NaCl to 1 M, and the final precipitate was dissolved in 0.5 M acetic acid and dialyzed against 0.1 M acetic acid.

All collagen solutions were aliquoted and stored at −20 °C until use. Protein concentrations were determined using Quantipro BCA kit (Sigma). Denatured jellyfish collagen was obtained by heating the solutions (60 °C, 20 min). Acid-soluble rat type I collagen was used as a control, and was purified as previously described [40]. Bovine pepsinized collagen was purchased from BD Bioscience.

Extracts submitted to collagenase digestion were dialyzed against 50 mM Tris-HCl, 0.2 M NaCl, CaCl_2_, pH 7.6. Then, collagenase (Advanced Biofacture) was added at 50 U/mL in the presence of 10 mM *N*-ethyl-maleimide, and samples were incubated for 5 h at 37 °C.

### 3.2. Ion-Exchange Chromatography

CM-cellulose (Whatman, CM52) for ion-exchange chromatography was prepared following the manufacturer’s instructions. Chromatography of native or denatured collagens was performed essentially as described elsewhere [19,41]. Briefly, native or denatured collagens in sodium acetate buffer, pH 4.8 (0.06 M or 0.02 M, respectively), were applied to a column of CM-cellulose. Collagens were eluted in sodium acetate buffer with a linear gradient of 0–0.5 M NaCl and 0–0.15 M NaCl for native and denatured collagens, respectively.

### 3.3. Solid-Phase Binding Assay for Heparin Binding

Solid-phase binding assays were performed to detect heparin-collagen interaction as described previously [31]. Briefly, 96-well plates (Nunc Maxisorp) were coated overnight with bovine or jellyfish collagen (5 μg/mL) at 4 °C, and subsequently saturated with T-PBS-BSA (PBS, 0.05% Tween 20, 1% Bovine Serum Albumin). Heparin-Albumin-Biotin at 5 μg/mL (Sigma) was added to the wells for 2 h, Binding was visualized by adding peroxidase-conjugated streptavidin (Sigma), H_2_O_2_ and ABTS (2,2-azino-bis(3-ethylbenthiazoline-6-sulfonic acid)), and by measure of the absorbance at 415 nm.

### 3.4. Heparin-Affinity Chromatography

Affinity chromatography of native or denatured jellyfish collagens was carried out on Sepharose-6 fast flow (GE Healthcare). Collagen samples were loaded onto the heparin-Sepharose column equilibrated with TBS (Tris-Buffered Saline; 50 mM Tris-HCl pH 7.5, 200 mM NaCl) and were eluted with a 0–0.5 M linear gradient of NaCl.

### 3.5. Cross-Linking of Jellyfish Collagen

Cross-linking of jellyfish collagen was realized using the EDC-NHS (1-ethyl-3-(3-dimethylaminopropyl)carbodiimide hydrochloride/*N*-hydroxysuccinimide) method developed by Olde Damink *et al.* [42]. Jellyfish collagen solutions (0.3 mg/mL in 0.1 M acetic acid) were mixed with an aqueous solution of EDC-NHS (2:1 molar ratio). Different collagen/EDC-NHS ratios (w/w, 1:1 to 1:11) were made. Samples were incubated overnight at room temperature and the reaction was stopped by addition of Tris-HCl (pH 7.4). Collagen cross-linking was assessed by SDS-PAGE.

### 3.6. Circular Dichroism

Triple helical conformation and thermal stability of jellyfish collagen were examined by acquisition of CD (Circular Dichroism) spectra. Measurements were done with a Chirascan circular dichroism spectrometer (Applied Photophysics) using a quartz cell with 0.2 cm optical path length. Spectra were collected from 180 to 260 nm. Data points for the thermal unfolding curves were recorded at 220 nm through 0.5 °C/min ramps (from 22 to 40 °C).

### 3.7. Cell Culture

Cell lines were obtained from ATCC, and human fibroblasts were a generous gift of O. Damour (Banque de cellules des Hospices Civils de Lyon, France). Cells were maintained at 37 °C in Dulbecco’s modified Eagle’s medium (DMEM, PAA Laboratories) supplemented with 10% fetal calf serum (FCS, PAA Laboratories) and 50 μg/mL gentamycin (Euromedex) in a 5% CO_2_ atmosphere.

### 3.8. Cell Cytotoxicity

Microtiter plates (96-well, Corning) were coated overnight at 4 °C with 50 μg/mL collagen solutions. Controls consisted in uncoated wells. Wells for each condition were done in triplicates. Cells were then added at 50,000 cells per well and incubated at 37 °C for 2 or 8 days. Viable cells were detected by adding MTT (3-(4,5-Dimethylthiazol-2-yl)-2,5-diphenyltetrazolium bromide) to a final concentration of 0.5 mg/mL for 2 h at 37 °C. The medium was removed from wells, and cells were resuspended in 10% TritonX-100 and 0.1 M HCl to dissolve formazan crystals present in viable cells. The absorbance was measured at 570 nm.

### 3.9. Cell Adhesion Assay

Cell adhesion to collagen adsorbed to microtiter plates was performed as previously described [43]. Briefly, 96-well plates (Nunc Maxisorp) were coated overnight at 4 °C with native collagens or at 37 °C with denatured collagens. Dose-response curves were obtained from coating with dilution series of collagen solutions. Wells were then saturated with 1% BSA. Cells suspended in serum-free medium were added to the wells (30,000 cells per well) and incubated for 30 min to 1 h at 37 °C. Non-adherent cells were removed, and adherent cells were fixed with 10% glutaraldehyde. Cells were stained with crystal violet, and the absorbance read at 570 nm. Inhibition studies were performed by using coating concentrations of 20 μg/mL for jellyfish collagen and 5 μg/mL for rat collagen. These concentrations used in the experiments were determined by dose-response studies. The putative inhibitor (antibody or heparin) was added to the cell suspension before distribution into the wells. Heparin (Sigma) was used at 10 µg/mL. Antibody against β1-integrin, clone AIIB2 obtained from Developmental Studies Hybridoma Bank (University of Iowa), was used at 10 µg/mL. Anti-αVβ3 (Chemicon MAB 1976) was diluted to 5 µg/mL The data points are expressed as means of triplicates, and each experiment was repeated a minimum of three times.

### 3.10. Immunofluorescence

Detection of cell-matrix adhesions was performed by immunolabeling of vinculin. Glass coverslips were coated with collagens at 5 μg/cm^2^. Cells were suspended in serum free medium and allowed to adhere to coverslips for 1 hour at 37 °C. After a brief rinsing in PBS (Phosphate-Buffered Saline), cells were fixed with 2.5% paraformaldehyde in PBS, and permeabilized with 0.1% Triton X-100 in PBS. Saturation of non-specific binding sites was performed by 1% BSA in PBS, before adding the primary antibody directed against vinculin, mouse anti-human vinculin (Chemicon MAB3574) diluted to 1 μg/mL in PBS. Alexa Fluor 546 goat anti-mouse (Invitrogen) diluted to 1 μg/mL in PBS was used as secondary antibody. Observation was performed with a Zeiss Axioplan epifluorescence microscope equipped with a Coolsnap fx digital camera (Roper scientific).

### 3.11. Statistical Analysis

All data are shown as mean ± Standard Deviation. Experiments had 3–5 biological replicates unless otherwise noted. For normal distribution data values, the statistical significance depicted was assessed by Student’s *t*-tests. *p*-values assess statistical significance between different treatments.

## 4. Conclusions

In this study, we have shown that the jellyfish species *R. pulmo* can be used as a natural marine source of collagens. Hence, *R. pulmo* collagen presents comparable biological impact on human cells than mammalian type I collagen tested by cytotoxicity and adhesion assays. Further investigations on the mechanisms of cell interaction led us to the conclusion that both integrins and heparan-sulfate receptors of human cells are able to recognize jellyfish collagen. Moreover, cells form focal adhesions similar to that observed on mammalian collagens, when they are plated on jellyfish collagen. These results suggest that, after *in vivo* implantation, jellyfish collagen would be able to induce similar responses in terms of cell adhesion, proliferation or migration. Considering the bioavailability of jellyfish collagen and its biological properties, this marine material is a good candidate for replacing bovine or human collagen in selected biomedical applications.

## Figures and Tables

**Table 1:** Yield of collagen after pepsin extraction. Values are indicated as mg of collagen per gram of wet tissue. Each extraction was performed from at least 10 g of tissue (wet weight) suspended in 10 mL of extraction solution/g of tissue.

**Species, organ**	**Collagen (mg/g)**
*Rhizostoma pulmo*, umbrella	0.83 to 3.15 (3 animals)
*Rhizostoma pulmo*, oral arms	2.61 to 10.3 (5 animals)
*Cotylorhiza tuberculata*, umbrella	0.453 (1 animal)
*Cotylorhiza tuberculata*, oral arms	1.94 (1 animal)
*Pelagia noctiluca*, whole body	0.074 (1 animal)
*Aurelia aurita*, whole body	0.0079 (1 animal)
